# Autotaxin levels in serum and bronchoalveolar lavage fluid are associated with inflammatory and fibrotic biomarkers and the clinical outcome in patients with acute respiratory distress syndrome

**DOI:** 10.1186/s40560-021-00559-3

**Published:** 2021-06-15

**Authors:** Lijuan Gao, Xiaoou Li, Hao Wang, Yue Liao, Yongfang Zhou, Ke Wang, Jun Hu, Mengxin Cheng, Zijian Zeng, Tao Wang, Fuqiang Wen

**Affiliations:** 1 Division of Pulmonary Disease, State Key Laboratory of Biotherapy of China, and Department of Respiratory and Critical Care Medicine, West China Hospital of Sichuan University, Guoxuexiang 37, Chengdu, 610041 Sichuan China; 2 Department of Critical Care Medicine, West China Hospital of Sichuan University, Guoxuexiang 37, Chengdu, 610041 Sichuan China

**Keywords:** Autotaxin, Acute respiratory distress syndrome, Prognosis, Mortality, Biomarker

## Abstract

**Background:**

Autotaxin (ATX) is a secreted glycoprotein that is widely present in extracellular biological fluids and has been implicated in many inflammatory and fibrotic diseases. However, the clinical impact of the release of ATX in patients with acute respiratory distress syndrome (ARDS) remains unclear.

**Methods:**

Serum and bronchoalveolar lavage fluid (BALF) levels of ATX, interleukin (IL)-6, IL-8, tumor necrosis factor (TNF)-α, matrix metalloproteinase (MMP)-7, fibronectin, oncostatin M (OSM), and SPARC (secreted protein acidic and rich in cysteine) were collected from 52 patients with ARDS within 24 h of diagnosis. All cytokines were measured by Magnetic Luminex Assay. BALF albumin (BA) and serum albumin (SA) were measured by enzyme-linked immunosorbent assay.

**Results:**

Serum ATX, MMP-7, and BALF IL-8 levels were significantly higher in patients who did not survive than in those who survived up to 28 days after diagnosis of ARDS (*P* < 0.05). BALF and serum ATX levels were correlated with IL-6, IL-8, and MMP-7 levels in BALF and serum, respectively. In addition, BALF ATX was positively correlated with BALF TNF-α, fibronectin, OSM, and SPARC as well as the BA/SA ratio, while serum ATX was correlated with severity of illness based on the SOFA score and PaO_2_/FIO_2_ ratio. Furthermore, serum ATX was better able to predict 28-day ARDS-related mortality (area under the curve 0.744, *P* < 0.01) than the SOFA score, APACHE II score, or PaO_2_/FIO_2_ ratio. Serum ATX independently predicted mortality in a univariate Cox regression model (*P* < 0.0001).

**Conclusion:**

The serum ATX level is a potential prognostic biomarker in patients with ARDS. BALF ATX is associated with pulmonary biomarkers of inflammation and fibrosis, suggesting a role of ATX in the pathogenesis of ARDS.

**Supplementary Information:**

The online version contains supplementary material available at 10.1186/s40560-021-00559-3.

## Background

Acute respiratory distress syndrome (ARDS) is a common and fatal complication of critical illness and is characterized by diffuse interstitial inflammation, non-cardiogenic pulmonary edema, and arterial hypoxemia [1, 2]. Despite decades of research effort, the mortality rate remains high in the range of 35–46% [3], particularly in patients who present with a fibroproliferative lung response [4]. An increasing body of research has demonstrated robust collagen synthesis in the lungs of patients with ARDS as early as 24 h after onset of the illness [5, 6]. Histological assessment of the lungs in patients with ARDS has clearly demonstrated that fibroproliferation is present early in a substantial proportion of patients [7]. At present, there is no therapy that specifically targets the dysregulated response that ultimately leads to respiratory failure. Therefore, understanding early signals that predict the long-term outcome in patients with ARDS may prove beneficial for predicting the need for more aggressive treatment and to identify novel therapeutic strategies.

Autotaxin (ATX) is a secreted glycoprotein [8] that catalyzes the hydrolysis of lysophosphatidylcholine to lysophosphatidic acid (LPA) [9, 10]. LPA plays an important role in many inflammatory diseases, including in vascular homeostasis, skeletal and stromal remodeling, lymphocyte trafficking, and immune regulation [11]. ATX is present in a variety of tissues, including adipose tissue, the central nervous system, reproductive organs, lung, kidney, pancreas, and biological fluids, including blood, cerebrospinal fluid, and bronchoalveolar lavage fluid (BALF) [12–14]. ATX expression is reportedly increased in patients with chronic liver disease and a shorter overall survival time [15] and in synovial fibroblasts from patients with rheumatoid arthritis in comparison with those from healthy controls [16]. Elevated ATX activity in plasma has recently been demonstrated to predict higher 30-day mortality in patients with sepsis [17]. Oikonomou et al. detected increased concentrations of ATX in a murine model of pulmonary fibrosis and in fibrotic human lungs, and genetic deletion of ATX from bronchial epithelial cells or macrophages and pharmacological inhibition of ATX can attenuate the severity of disease [18, 19]. GLPG1690, an ATX inhibitor, was reported to improve the prognosis by attenuating the decline in forced vital capacity in patients with idiopathic pulmonary fibrosis in a phase IIa randomized placebo-controlled trial [20]. However, the clinical significance of ATX in patients with ARDS remains unclear.

The objective of this study was to determine the link between ATX and ARDS and to identify the clinical value of ATX as a molecular biomarker of the status and risk of subsequent mortality in patients with ARDS by measuring ATX levels in BALF and serum and assessing the correlation of these levels with inflammatory and fibrotic indicators, disease severity, and patient outcomes.

## Methods

### Study design and subjects

This single-center cohort study was performed in the Department of Critical Care Medicine, West China Hospital, Sichuan University. The study protocol was designed according to the requirements of the Chinese Guidelines for Good Clinical Practice and was approved by the Ethics Committee of West China Hospital, Sichuan University (approval number: 2017.195). The study participants were patients aged > 18 years with a diagnosis of ARDS based on the Berlin criteria [21] who were treated at our institution between September 2017 and August 2018. Informed written consent was obtained from the legal representatives of all patients. Severity of illness was determined after the onset of ARDS using the Acute Physiology and Chronic Health Evaluation (APACHE) II and Sequential Organ Failure Assessment (SOFA) scores.

### Data extraction, collection of samples, and outcome measures

Demographic and baseline characteristics, including age, sex, and body mass index (BMI) were collected for all study participants. BALF and blood samples were obtained within 24 h of diagnosis of ARDS. The outcome (mortality within 28 days) was recorded.

BALF samples were obtained by fiberoptic bronchoscopy (with 100 mL of 0.9% saline solution sequentially instilled and suctioned in 20 mL portions) from a subsegment of the right middle lobe of the lung and recovered at a rate of 30–50% according to the procedure described in the 2012 American Thoracic Society guidelines [22]. After collecting BALF into tubes, the fluid retrieved was filtered through sterile gauze and centrifuged at 1000×*g* for 10 min at 4 °C to remove mucus and cells. The supernatants were aliquoted and frozen at −80 °C until analysis. Blood was collected into a vacutainer tube, stored immediately at 4 °C, allowed to clot, and then centrifuged at 1000×*g* for 10 min at 4 °C. Multiple aliquots of serum were frozen at −80 °C until measurement.

### Quantification of cytokine and albumin levels

The concentrations of BALF and serum ATX, interleukin (IL)-6, IL-8, tumor necrosis factor (TNF)-α, matrix metalloproteinase (MMP)-7, fibronectin, oncostatin M (OSM), and SPARC (secreted protein acidic and rich in cysteine) were measured using a Human Magnetic Luminex Assay (R&D Systems, Minneapolis, MN, USA) on a Bio-Plex 200 suspension array system (Bio-Rad, Hercules, CA, USA). Serum and BALF albumin concentrations were determined by enzyme-linked immunosorbent assay using a human albumin ELISA kit (Develop; Donglin Sci & Tech Development Co., Ltd., Jiangsu, China) with a detection range of 0.156–110 mg/mL. The BALF albumin to serum albumin (BA/SA) ratio was calculated as an index of lung permeability. These examinations were performed according to the manufacturer’s instructions.

### Statistical analysis

After the normality test, the data were summarized as the mean and standard deviation or as the median (interquartile range). When comparing two groups, an independent samples *t* test was used to evaluate normally distributed data and the Mann-Whitney test to examine non-normally distributed data. Spearman correlation analysis was used to determine correlations among variables. The area under the receiver operating characteristic (ROC) curve was used to identify predictive values for surviving patients. Kaplan-Meier plots and log-rank tests were used to compare survival rates. The association between the serum ATX level and 28-day mortality was assessed using univariate and multivariate Cox regression analyses. All statistical analyses were performed using SPSS version 26.0 (IBM Corp., Armonk, NY, USA) and GraphPad Prism 8.0.1 (GraphPad Software Inc., La Jolla, CA, USA). Statistical significance was set at *P* < 0.05.

## Results

### Baseline characteristics of patients with ARDS

Fifty-two patients with a diagnosis of ARDS were enrolled in the study. The characteristics at enrollment and outcomes in the study population are summarized in Table 1. Twenty-one of the 52 patients enrolled died during their hospital stay or within 28 days of diagnosis of ARDS. Non-survivors were older than survivors but there was no significant between-group difference in sex or BMI. Pneumonia was the most common etiology of ARDS in both survivors and non-survivors. There were more patients with indirect ARDS (caused by surgery, sepsis, trauma, hemorrhagic shock, acute pancreatitis, extra-pulmonary tumor, cerebrovascular disease, or pesticide poisoning) than direct ARDS (caused by pneumonia or pulmonary contusion) in the non-surviving group. SOFA scores were higher in the non-surviving group than in the surviving group (12.95 ± 3.58 vs 9.61 ± 2.87, *P* = 0.010, Table 1). However, there was no significant difference in the PaO_2_/FIO_2_ ratio, APACHE II score, or the tidal volume, positive end-expiratory pressure, or pH values between survivors and non-survivors (Table 1). Furthermore, there was no significant difference in BALF or serum ATX according to whether ARDS was the result of direct or indirect lung injury (*P* > 0.05; Additional file 1: Fig. S1). Similarly, no difference in BALF or serum ATX was found between direct and indirect lung injury in either the surviving or non-surviving subgroups (*P* > 0.05, Additional file 1: Table S1). Moreover, non-survivors were significantly more likely to have impairment of the hepatic (*P* = 0.006), renal (*P* = 0.034), and cardiovascular (*P* = 0.002) systems as well as coagulation disorders (*P* = 0.003; Additional file 1: Table S2).

**Table 1:** Baseline characteristics of patients with ARDS

Variables	ARDS survivors (n = 31)	ARDS non-survivors (n = 21)	*P* value
Age, years	32.03 ± 22.12	55.76 ± 17.20	0.280
Male, sex – no. (%)	22 (70.97%)	17 (80.95%)	0.415
BMI, kg/m^2^	25.37 ± 3.08	25.58 ± 2.57	0.800
Cause of ARDS – no. (%)			0.158
Direct (intrapulmonary) lung injury	18 (58.06%)	8 (38.10%)	
Indirect (extrapulmonary) lung injury	13 (41.94%)	13 (61.90%)	
Risk factor for ARDS – no. (%)			0.347
Pneumonia	17 (54.84%)	6 (28.57%)	
Sepsis	6 (19.35%)	3 (14.29%)	
Surgery	3 (9.68%)	4 (19.05%)	
Pulmonary contusion	1 (3.23%)	2 (9.52%)	
Trauma	1 (3.23%)	2 (9.52%)	
Other	3 (9.68%)	4 (19.05%)	
PaO_2_/FIO_2_, mmHg	114.59 ± 46.26	114.46 ± 64.49	0.994
APACHE II score	17.39 ± 6.07	21.05 ± 6.98	0.050
SOFA score	9.61 ± 2.87	12.95 ± 3.58	0.010
Mechanical ventilation setting			
Tidal volume, mL	425.63 ± 49.74	433.26 ± 144.60	0.800
PEEP (cmH2O)	6.26 ± 3.41	7.11 ± 3.11	0.395
pH	7.38 ± 0.11	7.31 ± 0.13	0.062
Length of stay in ICU stay – days	13.74 ± 16.30	8.10 ± 8.00	0.148

Data are presented as the mean ± standard deviation or as the median (interquartile range). Statistical significance was set at *P* < 0.05. *APACHE*, Acute Physiology and Chronic Health Evaluation; *ARDS*, acute respiratory distress syndrome; *BMI*, body mass index; *FIO*_*2*_, fraction of inspired oxygen; *ICU*, intensive care unit; *PaO*_*2*_, arterial oxygen tension; *PEEP*, positive end-expiratory pressure; *SOFA*, Sequential Organ Failure Assessment

### Comparison of studied biomarkers between survivors and non-survivors

**Fig. 1:**
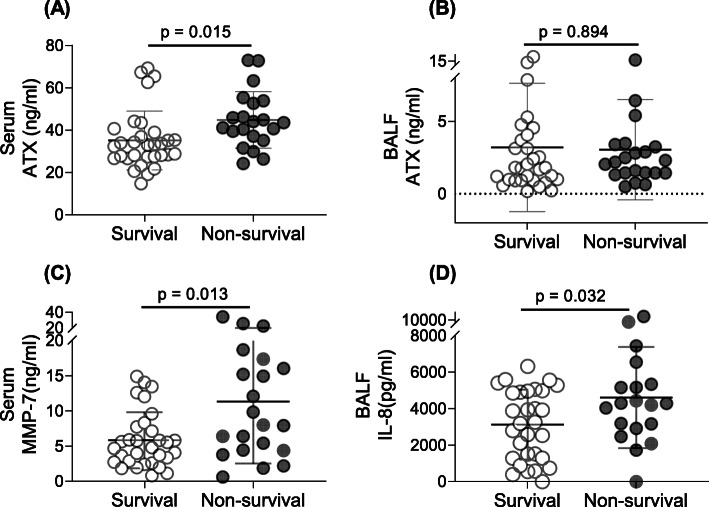
Comparison of ATX levels, serum IL-8, and BALF MMP-7 in survivors and non-survivors of ARDS. Serum ATX was significantly higher in non-survivors than in survivors (**A**). There was no significant difference in the BALF ATX level according to survival status in patients with ARDS (**B**). Serum MMP-7 (**C**) and BALF IL-8 (**D**) levels were significantly higher in non-survivors than in survivors. ARDS, acute respiratory distress syndrome; ATX, autotaxin; BALF, bronchoalveolar lavage fluid; IL-8, interleukin-8; MMP-7, matrix metalloproteinase-7

The values for the mediators investigated in the BALF and serum of survivors and non-survivors are shown in Table 2. The serum ATX level was markedly higher in non-survivors than in survivors (44.79 ± 13.38 ng/mL vs. 35.09 ± 13.89 ng/mL, *P* = 0.015, Table 2, Fig. 1A). There was no significant difference in BALF ATX between non-survivors and survivors (Fig. 1B). Serum MMP-7 and BALF IL-8 levels were higher in non-survivors than in survivors (Table 2; MMP-7, *P* = 0.013, Fig. 1C; IL-8, *P* = 0.032, Fig. 1D). There was no statistically significant between-group difference in BALF or serum levels of TNF-α, IL-6, SPARC, OSM, or fibronectin or in BALF MMP-7 or serum IL-8 levels (*P* > 0.05, Table 2).

**Table 2:** Comparison of biomarkers according to survival status

Biomarkers	BALF		Serum	
	ARDS survivors (n = 31)	ARDS non-survivors (n = 21)	ARDS survivors (n = 31)	ARDS non-survivors (n = 21)
ATX (ng/mL)	3.19 ± 4.43	3.04 ± 3.46	35.09 ± 13.89	44.79 ± 13.38*
IL-6 (ng/mL)	0.39 ± 0.56	0.61 ± 0.63	0.89 ± 3.83	0.97 ± 2.01
IL-8 (ng/mL)	3136.77 ± 1915.69	4618.65 ± 2776.99*	258.76 ± 735.09	314.47 ± 478.16
TNF-α (pg/mL)	13.27 (8.00-51.13)	35.56 (9.47-192.87)	12.04 (8.20-23.99)	14.72 (6.17-37.86)
MMP-7 (ng/mL)	34.03 ± 28.85	46.30 ± 43.18	4.81 (2.75-7.76)	8.02 (4.43-16.73) *
Fibronectin (ug/mL)	1.12 ± 1.74	1.12 ± 1.65	72.26 ± 111.07	78.21 ± 98.01
OSM (ng/mL)	0.93 ± 0.66	1.22 ± 0.76	3.65 ± 3.81	3.83 ± 5.25
SPARC (ng/mL)	19.50 ± 56.28	15.84 ± 37.57	1.33 ± 0.92	1.03 ± 0.12

Data are presented as the mean ± standard deviation or median (interquartile range). Statistical significance was set at *P*<0.05. *ARDS*, acute respiratory distress syndrome; *ATX*, autotaxin; *BALF*, bronchoalveolar lavage fluid; *IL-6*, interleukin-6; *IL-8*, interleukin-8; *MMP-7*, matrix metalloproteinase-7; *OSM*, oncostatin M; *SPARC*, secreted protein acidic and rich in cysteine; *TNF-α*, tumor necrosis factor-α *P<0.05 vs survivors

### Association of ATX with biomarkers of inflammation or fibrosis and the BA/SA ratio

Spearman correlation analysis was used to assess the correlation between ATX and ARDS-related biomarkers of inflammation and fibrosis (Table 3). BALF ATX levels were positively associated with IL-6, IL-8, TNF-α, MMP-7, fibronectin, OSM, and SPARC levels, while serum ATX levels were positively correlated with serum IL-6, IL-8, and MMP-7 levels. However, serum TNF-α, fibronectin, OSM, and SPARC levels were not significantly associated with serum ATX levels. A positive correlation was found between BALF levels of ATX and the BA/SA ratio. There was no correlation between the serum ATX level and the BA/SA ratio.

**Table 3:** Correlations between ATX and a panel of biomarkers

Biomarkers	BALF		Serum	
	Spearman’s rank (95% CI)	*P* value	Spearman’s rank (95% CI)	*P* value
IL-6	0.552 (0.297-0.734)	0.0001	0.511 (0.260-0.697)	0.0002
IL-8	0.289 (−0.003-0.536)	0.0460	0.313 (0.033-0.548)	0.0254
TNF-α	0.562 (0.331-0.729)	< 0.0001	0.245 (−0.038-0.492)	0.0803
MMP-7	0.446 (0.186-0.647)	0.0010	0.471 (0.219-0.664)	0.0004
Fibronectin	0.702 (0.516-0.825)	< 0.0001	0.060 (−0.224-0.335)	0.6714
OSM	0.508 (0.262-0.692)	0.0001	0.268 (−0.014-0.510)	0.0550
SPARC	0.620 (0.408-0.768)	< 0.0001	−0.078 (−0.351-0.207)	0.5828
BA/SA ration (‰)	0.658 (0.502-0.772)	< 0.0001	−0.014 (−0.237-0.210)	0.9024

*P* values less than 0.05 are statistically significant. *ARDS*, acute respiratory distress syndrome; *ATX*, autotaxin; *BA*, BALF albumin; *BALF*, bronchoalveolar lavage fluid; *CI*, confidence interval; *IL-6*, interleukin-6; *IL-8*, interleukin-8; *MMP-7*, matrix metalloproteinase-7; *OSM*, oncostatin M; *SA*, serum albumin; *SPARC*, secreted protein acidic and rich in cysteine; *TNF-α*, tumor necrosis factor-alpha

### Association of the ATX level with severity of ARDS

**Fig. 2:**
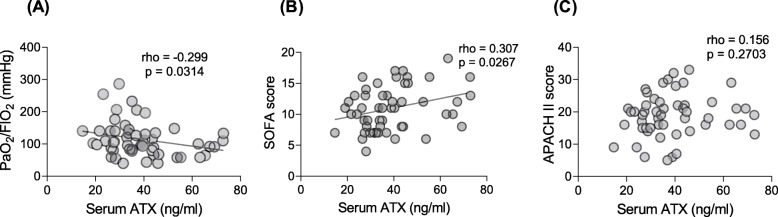
Correlation of serum ATX level with severity of ARDS. The serum ATX level showed a significant negative correlation with the PaO_2_/FIO_2_ ratio (**A**) and a positive correlation with the SOFA score (**B**) but not with the APACHE II score (**C**). APACHE, Acute Physiology and Chronic Health Evaluation; ARDS, acute respiratory distress syndrome; ATX, autotaxin; FIO_2_, fraction of inspired oxygen; PaO_2_, arterial oxygen tension; SOFA, Sequential Organ Failure Assessment

The relationships between ATX and markers of ARDS severity were analyzed using the Spearman correlation coefficient. Serum ATX levels were correlated with the PaO_2_/FIO_2_ ratio (rho −0.299, *P* = 0.0314, Fig. 2A) and SOFA score (rho 0.307, *P* = 0.0267, Fig. 2B). However, there was no statistically significant correlation between the BALF ATX level and the SOFA score or PaO_2_/FIO_2_ ratio (Table 4). Similarly, no relationship was found between BALF and serum ATX levels and APACHE II scores (Table 4; Fig. 2C).

**Table 4:** Correlation of BALF and serum ATX levels with SOFA and APACHE II scores and the PaO_2_/FIO_2_ ratio in patients with ARDS

Biomarkers	BALF ATX		Serum ATX	
	Spearman’s rank (95% CI)	*P* value	Spearman’s rank (95% CI)	*P* value
SOFA score	0.200 (−0.080-0.451)	0.1587	0.307 (0.029-0.541)	0.0267
APACHE II score	−0.019 (−0.301-0.266)	0.8951	0.156 (−0.131-0.418)	0.2703
PaO_2_/FIO_2_	−0.234 (−0.485-0.053)	0.0991	−0.299 (−0.5345-0.020)	0.0314

*P* values less than 0.05 are statistically significant. *APACHE*, Acute Physiology and Chronic Health Evaluation; *ATX*, autotaxin; *BALF*, bronchoalveolar lavage fluid; *CI*, confidence interval; *FIO*_*2*_, fraction of inspired oxygen; *PaO*_*2*_, arterial oxygen tension; *SOFA*, Sequential Organ Failure Assessment

### Association of the ATX level with outcome of ARDS

**Fig. 3:**
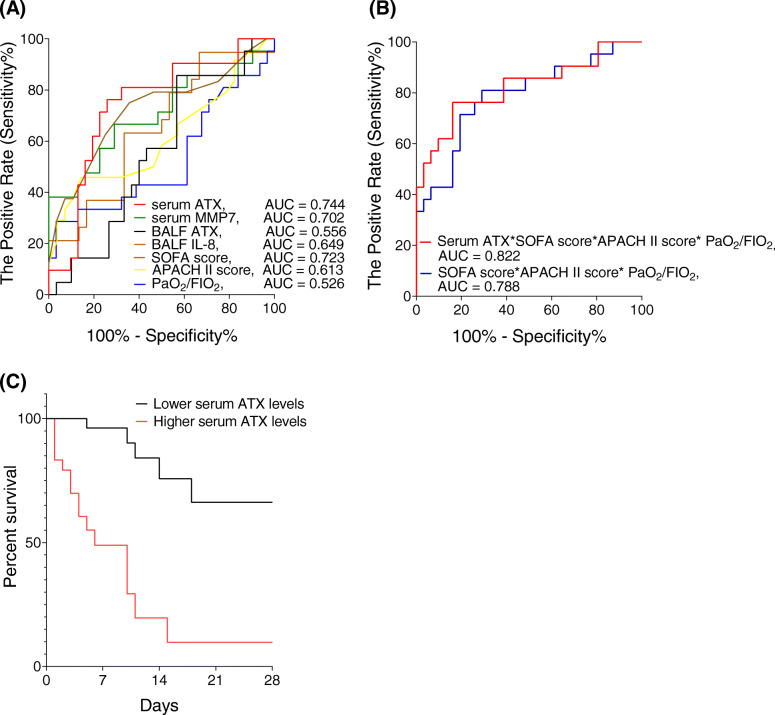
Ability of serum ATX to predict mortality in patients with ARDS. Receiver operating characteristic curve analysis of the ability of the serum ATX level to predict mortality in patients with ARDS was higher than that of serum MMP-7 (**A**), BALF IL-8 (**A**), the SOFA score (**A**), the APACHE II score (**A**), or the PaO_2_/FIO_2_ ratio (**A**). A combination of the serum ATX level and the SOFA score, APACHE II score, and PaO_2_/FIO_2_ ratio could help to predict the prognosis of ARDS (**B**). Kaplan–Meier curves show that the survival rate of patients stratified with a cutoff value of 36.96 ng/mL for serum ATX was significantly lower than that of patients with a lower ATX level (*P* < 0.0001, log-rank test) (**C**). APACHE, Acute Physiology and Chronic Health Evaluation; ARDS, acute respiratory distress syndrome; ATX, autotaxin; AUC, area under the receiver operating characteristic curve; BALF, bronchoalveolar lavage fluid; FIO_2_, fraction of inspired oxygen; IL-8, interleukin-8; MMP-7, matrix metalloproteinase-7; PaO_2_, arterial oxygen tension; SOFA, Sequential Organ Failure Assessment

In view of the relationship observed between the serum ATX level and the severity of ARDS, the ability of the serum ATX level to predict mortality was explored by ROC curve analysis. A serum ATX level with an area under the curve (AUC) of 0.744 (95% confidence interval [CI] 0.603–0.884, *P* = 0.0031, Fig. 3A) could predict 28-day mortality better than BALF IL-8 (AUC 0.649, 95% CI 0.490–0.808, *P* = 0.0811, Fig. 3A) or serum MMP-7 (AUC 0.702, 95% CI 0.548–0.856, *P* = 0.0142, Fig. 3A). Furthermore, using the SOFA score, APACHE II score, and PaO_2_/FIO_2_ ratio to separately predict 28-day mortality, we obtained respective AUC values of 0.723 (95% CI 0.580–0.867, *P* = 0.0059, Fig. 3A), 0.613 (95% CI 0.454–0.772, *P* = 0.1630, Fig. 3A), and 0.526 (95% CI 0.355–0.698, *P* = 0.7512, Fig. 3A), suggesting that the predictive ability of serum ATX was higher than that of the typical indicators associated with the severity of ARDS. Moreover, in combination with serum ATX, the combined AUC for the SOFA score, APACHE II score, and PaO_2_/FIO_2_ ratio increased from 0.788 (95% CI 0.658–0.918, *P* = 0.0005) to 0.822 (95% CI 0.697–0.946, *P* < 0.0001; Fig. 3B).

When the serum ATX level was used to predict the prognosis, ROC curve analysis identified a cutoff value of 36.96 ng/mL with sensitivity and specificity values of 76.19% (95% CI 0.55–0.89) and 74.19% (95% CI 0.57–0.86), respectively. The Kaplan-Meier survival curve showed that a higher serum ATX value was associated with a higher risk of death (*P* < 0.0001, Fig. 3C). The SOFA score was significantly higher in the subgroup with a higher serum ATX level (> 36.96 ng/mL) than in the subgroup with a lower serum ATX level (12.46 ± 3.66 vs. 9.68 ± 2.96, *P* = 0.004, Additional file 1: Table S4).

A univariate Cox regression model was built to assess the relationship between the serum ATX level and the outcome in patients with ARDS. Table 5 shows that the SOFA score (hazard ratio [HR] 1.234, 95% CI 1.084–1.404, *P* = 0.001), APACHE II score (HR 1.103, 95% CI 1.026–1.186, *P* = 0.008), and serum ATX level (HR 2.605, 95% CI 1.530–4.435, *P* < 0.0001) were independent predictors of 28-mortality in patients with ARDS. In multivariate analysis, only the APACHE II score (HR 1.098, 95% CI 1.017–1.186, *P* = 0.017, Table 5) and serum ATX level remained independently associated with 28-day mortality (per 20 ng/mL, HR 2.479, 95% CI 1.200–5.121, *P* = 0.014, Table 5).

**Table 5:** Cox proportional hazards models for prediction of 28-day mortality based on the ATX level and severity score

Variable	Univariate Cox model HR (95% CI)	*P* value	Multivariate Cox model HR (95% CI)	*P* value
SOFA (per point)	1.234 (1.084-1.404)	0.001	1.108 (0.938-1.307)	0.227
APACHE II (per point)	1.103 (1.026-1.186)	0.008	1.098 (1.017-1.186)	0.017
PaO_2_/FIO_2_ (per 10 mmHg)	0.987 (0.905-1.076)	0.767	0.993 (0.906-1.088)	0.880
BALF ATX (per 1 ng/mL)	1.040 (0.932-1.162)	0.482	1.004 (0.870-1.158)	0.956
Serum ATX (per 20 ng/mL)	2.605 (1.530-4.435)	< 0.0001	2.479 (1.200-5.121)	0.014

*P* values less than 0.05 are statistically significant. *APACHE*, Acute Physiology and Chronic Health Evaluation; *ATX*, autotaxin; *BALF*, bronchoalveolar lavage fluid; *CI*, confidence interval; *FIO*_*2*_, fraction of inspired oxygen; *HR*, hazard ratio; *PaO*_*2*_, arterial oxygen tension; *SOFA*, Sequential Organ Failure Assessment

## Discussion

To the best of our knowledge, this is the first study to demonstrate the potential value of ATX as a predictor of the prognosis in patients with ARDS. We investigated the differences in serum and BALF ATX levels between patients with ARDS who survived and those who did not and found significant differences in serum ATX, serum MMP-7, and BALF IL-8 levels between survivors and non-survivors. More importantly, we demonstrated that serum ATX levels were independently associated with the SOFA score and PaO_2_/FIO_2_ ratio, which are widely used indicators of ARDS severity, and that there was a relationship between an elevated serum ATX level and a higher risk of mortality in patients with ARDS. These findings suggest that an increased serum ATX level is associated with more clinically severe ARDS and an adverse outcome. Furthermore, we found that ATX levels were associated with biomarkers of inflammation and fibrosis, including IL-6, IL-8, TNF-α, MMP-7, OSM, SPARC, and fibronectin. On balance, these results demonstrate the predictive significance of ATX in ARDS and may help to identify novel therapeutic strategies for patients with this disease.

Some investigations during the past decade have addressed the potential role of ATX in several pulmonary diseases. Elevated ATX staining and activity were detected in biopsies and serum samples from patients with lung cancer [23] and there was an increase in BALF ATX levels in patients with asthma [24] and idiopathic pulmonary fibrosis (IPF) [18]. Furthermore, there was a report of an increased BALF ATX level in a murine model of LPS-induced acute lung injury [25]. However, the clinical significance of ATX in patients with ARDS has not been investigated in detail. As is well known, the pathophysiology of ARDS is multifactorial and includes pulmonary inflammation, barrier disruption, interstitial and airspace edema, cell injury, and cell death. Lung injury can increase as the disease progresses through the exudative and proliferative phases, leading to a fibrotic phase and worsening lung function [1]. As previously described, ATX, a secreted lysophospholipase D that generates the bioactive lipid LPA, has been confirmed to play a pathological role in pulmonary inflammation in asthma [24] and lung fibrosis in IPF [18]. Therefore, we sought to explore the relationship between ATX and a panel of biomarkers previously implicated in the pathogenesis of ARDS. It has been demonstrated that TNF-α, IL-6, and IL-8 are involved in the pathogenesis of inflammation in ARDS [26]. Moreover, MMP-7 has been reported to direct and confine neutrophil influx to sites of injury by mediating shedding of syndecan-1 complexes from the mucosal surface in acute lung injury [27]. In this study, BALF and serum ATX levels were closely correlated with those of TNF-α, IL-6, IL-8, and MMP-7, indicating that ATX may play a role in the regulation of pulmonary inflammation in ARDS. Furthermore, fibronectin, OSM, and SPARC have been reported to participate in pathological situations such as tissue remodeling and pulmonary fibrosis in ARDS [28–32]. Grenier et al. reported that OSM was released by alveolar polymorphonuclear neutrophils in acute lung injury [33]. Furthermore, OSM can induce production of collagen as well as proliferation and reduced apoptosis in human lung fibroblasts [34] depending on the activation status of STAT3 [35]. Meanwhile, single-cell RNA sequencing identified increased gene expression of SPARC in the monocytes of patients with ARDS [29], and overexpression of SPARC in IPF potentially drove tissue fibrosis via induction of PAI-1 expression, leading to a pool of myofibroblasts that are resistant to apoptosis and, consequently, a dysregulated milieu in the extracellular matrix [36]. In our study, all mediators of fibrosis in BALF were significantly associated with BALF ATX, indicating that ATX may have a role in the pulmonary fibrosis component of ARDS. Overall, ATX may play an important role in the regulation of inflammation and fibrosis in ARDS, but the specific mechanism needs to be explored in future studies.

Various scoring systems, including SOFA, APACHE II, and the PaO_2_/FIO_2_ ratio, have been widely used in clinical practice to predict the outcome of ARDS. However, these scoring systems have several shortcomings in terms of predicting disease progression because of the interobserver variability arising from complicated calculations and subjective judgments [37, 38]. Therefore, there is a need for biomarkers that have potential prognostic ability in ARDS and can be easily obtained. In this study, a significantly elevated serum ATX level was found more often in non-survivors than in survivors, suggesting that serum ATX is associated with the progression of ARDS. Significant correlations were observed between the serum ATX level and the SOFA score and PaO_2_/FiO_2_ ratio. However, the BALF ATX concentration was not significantly different between patients who survived and those who did not and was not correlated with the SOFA score or the PaO_2_/FiO_2_ ratio. Furthermore, there was no significant difference in the serum or BALF ATX level according to whether lung injury was direct or indirect in either the surviving or non-surviving subgroups, suggesting that the cause of ARDS (direct or indirect lung injury) is not the main factor affecting the prognostic ability of serum and BALF ATX. One potential reason may be that the serum ATX level, but not BALF ATX level, was correlated with the SOFA score. Given that the presence of comorbidities and multiorgan dysfunction are associated with mortality in ARDS, the serum ATX level may be more closely related to the severity of multiorgan dysfunction, and thereby help to predict the likelihood of mortality in patients with ARDS. In addition to the higher serum ATX level and higher SOFA score, there were significantly more liver, renal, cardiovascular, and coagulation injury in non-survivors. Previous studies have demonstrated an increase in the serum ATX level in patients with kidney or liver disease [39, 40]. In order to analyze the factors contributing to the elevated serum ATX level, we performed a binary logistic regression analysis of the association of serum ATX level (low or high based on a cutoff value of 36.96 ng/mL) with the severity of lung injury (based on a PaO_2_/FIO_2_ ratio > 200 mmHg or ≤ 200 mmHg), multiorgan damage (with or without liver, renal, cardiovascular, nervous system, or coagulation injury), and the etiology of ARDS (direct or indirect injury). We found that the severity of lung injury was significantly correlated with the serum ATX level (*P* = 0.029, Additional file 1: Table S3), indicating that lung injury may be one of the main causes of the elevated serum ATX. Cardiovascular injury may also have played a role in the change in serum ATX level in this study (*P* = 0.025) and warrants more clinical and mechanistic studies in the future.

Furthermore, we found that the prognostic value of serum ATX was higher than that of the biomarkers previously reported in ARDS, including serum MMP-7 and BALF IL-8, and the common indicators of ARDS (the SOFA score, APACHE II score, and PaO_2_/FIO_2_ ratio). In combination with serum ATX, the predictive ability of these three typical indicators combined can be increased. Serum ATX levels can be easily and quickly obtained in clinical settings. For risk assessment, we found that a higher SOFA score could predict higher mortality in patients with ARDS, which is in agreement with previous findings [41, 42]. In our study, patients with ARDS and a serum ATX level > 36.96 ng/mL had a higher SOFA score and a higher 28-day mortality rate according to the Kaplan-Meier survival curve. Notably, the serum ATX level was significantly associated with the risk of ARDS-related death in a univariate Cox regression model, indicating that each 20-ng/mL increment in the serum ATX level increased the risk of death from ARDS by nearly threefold. The multivariate Cox regression model did not identify the PaO_2_/FIO_2_ ratio or the SOFA score as a significant prognostic factor. One potential reason for this finding might be the small sample size, which limited our ability to assess the predictive ability of the PaO_2_/FIO_2_ ratio and SOFA score in the study. Another reason may be that the PaO_2_/FIO_2_ ratio mainly reflects the severity of local lung injury and the SOFA score focuses on six organic variables at the time of grading, whereas the APACHE II contains features reflecting both acute and chronic conditions and is more comprehensive when estimating risk [43]. Therefore, when used to predict mortality in patients with ARDS, APACHE II performed better in the multivariate Cox model. No correlation was found between the ATX level and the APACHE II score. The AUC of APACHE II for predicting 28-day mortality was only 0.613 (*P* > 0.05, Fig. 3A). One possible reason for this finding is that ATX may be more sensitive than the APACHE II score for predicting the outcome of patients with ARDS. Overall, our results suggest that ATX may be a strong independent predictor that can be used to supplement SOFA and APACHE II scores and the PaO_2_/FIO_2_ ratio when evaluating the severity of ARDS and the likely outcome for the patient.

This study has several limitations that need to be overcome in future research. First, the small sample size might have limited our ability to analyze the ATX level in different ARDS phenotypes. Second, we only measured ATX levels in the first 24 h after enrollment. Therefore, the relationship between the ATX level and progression of pulmonary fibrosis in patients with ARDS was not followed by imaging or pathological observation. Future studies with larger sample sizes and longer follow-up durations are needed to further explore the role of ATX in the pathogenesis of ARDS.

## Conclusions

Overall, in this study, the ATX level showed a good correlation with a panel of biomarkers previously associated with pulmonary inflammation and fibrosis. The serum ATX level was significantly correlated with disease severity and appeared to have good prognostic value when applied alone or in combination with the current methods used to predict the outcome of ARDS (SOFA, APACHE II, and PaO_2_/FIO_2_). Our findings strongly suggest that the serum ATX level can be used to predict the prognosis in patients with ARDS. This study also advances our understanding of the mechanisms underlying the regulation of pulmonary inflammation and fibrosis in ARDS. Further clinical and mechanistic studies are warranted to elucidate the specific role of ATX in the pathogenesis of ARDS.

## Supplementary Information

**Additional file 1.** Figure S1. Comparison of ATX levels in patients with ARDS according to whether lung injury was direct or indirect. Table S1. Comparison of ATX levels according to whether lung injury was direct or indirect in either survivors or non-survivors of ARDS. Table S2. Information about organ injury according to survival status. Table S3. Binary logistic regression analysis of low and high serum ATX levels. Table S4. Comparison of disease severity according to the serum ATX level.

## Data Availability

The datasets used and/or analyzed during the current study are available from the corresponding author on reasonable request.

## Abbreviations

APACHE: Acute Physiology and Chronic Health Evaluation
ARDS: Acute respiratory distress syndrome
ATX: Autotaxin
AUC: Area under the receiver-operating characteristic curve
BA: BALF albumin
BALF: Bronchoalveolar lavage fluid
BMI: Body mass index
CI: Confidence interval
FIO_2_: Fraction of inspired oxygen
HR: Hazard ratio
IPF: Idiopathic pulmonary fibrosis
IL: Interleukin
LPA: Lysophosphatidic acid
MMP-7: Matrix metalloproteinase-7
OSM: Oncostatin M
PaO_2_: Arterial oxygen tension
ROC: Receiver operating characteristic
SA: Serum albumin
SOFA: Sequential Organ Failure Assessment
SPARC: Secreted protein acidic and rich in cysteine
TNF-α: Tumor necrosis factor-alpha
