# Supplementary material for: Autotaxin levels in serum and bronchoalveolar lavage fluid are associated with inflammatory and fibrotic biomarkers and the clinical outcome in patients with acute respiratory distress syndrome
Source: J Intensive Care. 2021 Jun 15;9:44. doi: 10.1186/s40560-021-00559-3 (PMC8207767; doi:10.1186/s40560-021-00559-3)
Supplement: Supplementary file 1 — Additional file 1. Figure S1. Comparison of ATX levels in patients with ARDS according to whether lung injury was direct or indirect. Table S1. Comparison of ATX levels according to whether lung injury was direct or indirect in either survivors or non-survivors of ARDS. Table S2. Information about organ injury according to survival status. Table S3. Binary logistic regression analysis of low and high serum ATX levels. Table S4. Comparison of disease severity according to the serum ATX level. [file 40560_2021_559_MOESM1_ESM.docx]

**Figure S1. Comparison of ATX levels in patients with ARDS according to whether lung injury was direct or indirect.**
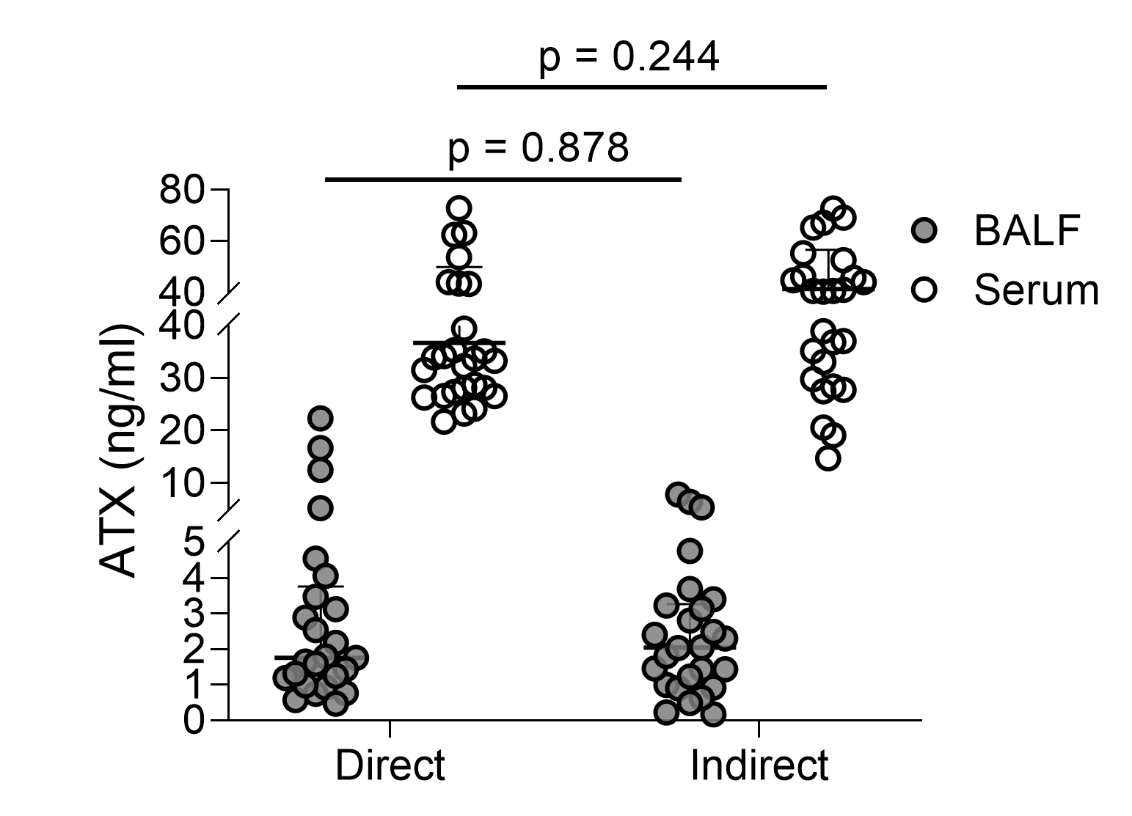


**Table S1** **Comparison of ATX levels according to whether lung injury was direct or indirect in either survivors or non-survivors of ARDS**

| ATX levels | Survivors | | | Non-survivors | | |
| --- | --- | --- | --- | --- | --- | --- |
|  | Direct lung injury  (n=18) | Indirect lung injury  (n=13) | *P* value | Direct lung injury (n=8) | Indirect lung injury (n=13) | *P* value |
| BALF ATX (ng/mL) | 3.86±5.57 | 2.32±2.15 | 0.352 | 3.79±5.28 | 2.58±1.74 | 0.451 |
| Serum ATX (ng/mL) | 33.24±9.46 | 37.65±18.54 | 0.443 | 44.36±17.69 | 45.05±10.75 | 0.912 |

Data are presented as the mean ± standard deviation. ARDS, acute respiratory distress syndrome; BALF, bronchoalveolar lavage fluid.

**Table S2** **Information about organ injuries according to survival status**

| Organ | ARDS survivors  (n=31) | ARDS non-survivors (n=21) | *P* value |
| --- | --- | --- | --- |
| Respiratory, dysfunction- no. (%) | 31 (100%) | 21 (100%) | - |
| Liver, dysfunction- no. (%) | 10 (32.26%) | 15 (71.43%) | 0.006 |
| Renal, dysfunction- no. (%) | 20 (64.52%) | 19 (90.48%) | 0.034 |
| Cardiovascular, dysfunction- no. (%) | 15 (48.39%) | 19 (90.48%) | 0.002 |
| Nervous, dysfunction- no. (%) | 27 (87.10%) | 18 (85.71%) | 0.886 |
| Coagulation, dysfunction- no. (%) | 9 (29.03%) | 15 (71.43%) | 0.003 |

Categorical variables are presented as the number (percentage). Statistical significance was set at P<0.05. ARDS, acute respiratory distress syndrome.

**Table S3 Binary logistic regression analysis of low and high serum ATX levels**

| Factor | Odds Ratio (95%CI) | *P* value |
| --- | --- | --- |
| PaO_2_/ FIO_2_  >200mmHg  ≤200mmHg | 1 (Reference)  4.08 (1.16−14.35) | 0.029 |
| Liver  Non-dysfunction  Dysfunction | 1 (Reference)  2.63 (0.54−12.90) | 0.233 |
| Renal  Non-dysfunction  Dysfunction | 1 (Reference)  1.86 (0.29−12.12) | 0.517 |
| Cardiovascular  Non-dysfunction  Dysfunction | 1 (Reference)  7.99 (1.31−48.97) | 0.025 |
| Nervous  Non-dysfunction  Dysfunction | 1 (Reference)  0.117 (0.01−1.93) | 0.134 |
| Coagulation  Non-dysfunction  Dysfunction | 1 (Reference)  1.26 (0.24−6.53) | 0.784 |
| Cause of ARDS  Direct lung injury  Indirect lung injury | 1 (Reference)  3.02(0.64−14.35) | 0.165 |

Statistical significance was set at P<0.05. ARDS, acute respiratory distress syndrome; ATX, autotaxin; FiO_2_, fraction of inspired oxygen; PaO_2_, arterial oxygen tension.

**Table S4 Comparison of disease severity according to serum ATX level.**

| Variables | Lower ATX level in serum  (n=28) | Higher ATX level in serum  (n=24) | *P* value |
| --- | --- | --- | --- |
| SOFA score | 9.68±2.96 | 12.46±3.66 | 0.004 |
| PaO_2_/FiO_2_, mmHg | 127.67±59.15 | 99.22±42.98 | 0.056 |
| APACHE Ⅱ score | 17.64±5.91 | 20.29±7.26 | 0.153 |

Data are presented as the mean ± standard deviation. Statistical significance was set at P<0.05. ATX, autotaxin; APACHE, Acute Physiology and Chronic Health Evaluation; ARDS; FiO_2_, fraction of inspired oxygen; PaO_2_, arterial oxygen tension; SOFA, Sequential Organ Failure Assessment.
